# Heat shock protein 70 expression, keratin phosphorylation and Mallory body formation in hepatocytes from griseofulvin-intoxicated mice

**DOI:** 10.1186/1476-5926-3-5

**Published:** 2004-08-12

**Authors:** Michel Fausther, Louis Villeneuve, Monique Cadrin

**Affiliations:** 1 Département de chimie-biologie, Université du Québec à Trois-Rivières, 3351 boulevard des Forges, C.P. 500, Québec, Trois-Rivières, Canada G9A 5H7

## Abstract

**Background:**

Keratins are members of the intermediate filaments (IFs) proteins, which constitute one of the three major cytoskeletal protein families. In hepatocytes, keratin 8 and 18 (K8/18) are believed to play a protective role against mechanical and toxic stress. Post-translational modifications such as phosphorylation and glycosylation are thought to modulate K8/18 functions. Treatment of mouse with a diet containing griseofulvin (GF) induces, in hepatocytes, modifications in organization, expression and phosphorylation of K8/18 IFs and leads, on the long term, to the formation of K8/18 containing aggregates morphologically and biochemically identical to Mallory bodies present in a number of human liver diseases. The aim of the present study was to investigate the relationship between the level and localization of the stress inducible heat shock protein 70 kDa (HSP70i) and the level and localization of K8/18 phosphorylation in the liver of GF-intoxicated mice. The role of these processes in Mallory body formation was studied, too. The experiment was carried out parallely on two different mouse strains, C3H and FVB/n.

**Results:**

GF-treatment induced an increase in HSP70i expression and K8 phosphorylation on serines 79 (K8 S79), 436 (K8 S436), and K18 phosphorylation on serine 33 (K18 S33) as determined by Western blotting. Using immunofluorescence staining, we showed that after treatment, HSP70i was present in all hepatocytes. However, phosphorylated K8 S79 (K8 pS79) and K8 S436 (K8 pS436) were observed only in groups of hepatocytes or in isolated hepatocytes. K18 pS33 was increased in all hepatocytes. HSP70i colocalized with MBs containing phosphorylated K8/18. Phophorylation of K8 S79 was observed in C3H mice MBs but was not present in FVB/n MBs.

**Conclusions:**

Our results indicate that GF intoxication represents a stress condition affecting all hepatocytes, whereas induction of K8/18 phosphorylation is not occurring in every hepatocyte. We conclude that, *in vivo*, there is no direct relationship between GF-induced stress and K8/18 phosphorylation on the studied sites. The K8/18 phosphorylation pattern indicates that different cell signaling pathways are activated in subpopulations of hepatocytes. Moreover, our results demonstrate that, in distinct genetic backgrounds, the induction of K8/18 phosphorylation can be different.

## Background

Intermediate filaments (IFs) with microtubules and actin microfilaments are the major cytoskeletal components of most vertebrate cells [1-4]. IF proteins constitute a large family of proteins that is divided into five types [1,2]. The expression of the different IF proteins is differentiation and tissue specific [1,5]. Keratins expressed in epithelial cells, represent the largest and most complex subtype of IF proteins (more than 20 proteins)[2]. They are classified into two groups, the type I (acidic K9 to K20) and the type II (neutral-basic, K1 to K8), which form obligate heteropolymers composed of equimolar amounts of type I and type II keratins [2,6].

It is now generally accepted that, in multilayered epithelia, one of the function for keratins IFs is to protect the tissue from mechanical stress [7-9]. The first evidences for this function came from studies on epidermis, which showed that transgenic mice lacking epidermal keratins, or expressing mutated keratins, displayed blistering skin disease phenotypes, similar to human skin diseases such as epidermolysis bullosa simplex or epidermolytic hyperkeratosis [7,10,11].

As for epidermal keratins, the production of transgenic mice targeting K8 or K18 has been necessary to unravel the role of IFs in simple epithelium such as in the liver. In hepatocytes, K8/18 is the only keratin pair and thus both keratins are necessary to form an IF network. Transgenic mice expressing K8 or K18 carrying mutations that affect filament formation, develop mild hepatitis and display greater liver sensitivity to mechanical and toxic stress than wild type animals [12,13].

Recent studies from Ku et al. [14-16] have shown that mutations on K8/18 predispose to the development of liver disease in humans. Moreover, modifications in IF organization and the formation of keratin containing aggregates, named Mallory bodies (MBs), are observed in different liver diseases such as alcoholic hepatitis, Wilson's disease, Indian childhood cirrhosis and liver steatosis in obesity [17-21]. Other proteins, such as ubiquitin and the heat shock protein 70 kDa (HSP70), are also present in MBs and could play a role in their formation [22-24]. Taken together, these results support the hypothesis that keratins are necessary to preserve the hepatocytes integrity upon stressful conditions. It is still unclear how keratins accomplish these protective roles. Previous studies have shown that modifications in keratin phosphorylation are associated with various conditions such as mitosis, apoptosis and stress, suggesting a role for this post-translational modification in the modulation of keratin-related functions [25-27].

Long-term treatment of mice with a diet containing griseofulvin (GF) induces the development of an hepatitis associated with the formation of MBs, which are biochemically and morphologically similar to those found in humans [19,28]. This animal model constitutes a useful tool to investigate the keratin dynamics in the response of hepatocytes to the presence of a hepatotoxic agent.

In the present study, we investigated the effect of chronic GF intoxication on hepatocytes from C3H and FVB/n mouse strains. We monitored the expression of the stress inducible form of the heat shock protein 70 kDa (HSP70i) and the induction of K8/18 phosphorylation at specific sites: K8 on serine 79 (K8 S79), K8 on serine 436 (K8 S436), K18 on serine 52 (K18 S52) and K18 on serine 33 (K18 S33) (reviewed in [26,29]). We also examined the possible relationship between HSP70i expression and K8/18 phosphorylation, during the development of hepatitis and in MB formation.

## Results

### Induction of HSP70i and K8/18 phosphorylation upon GF-treatment in C3H and FVB/n mouse livers

**Figure 1:**
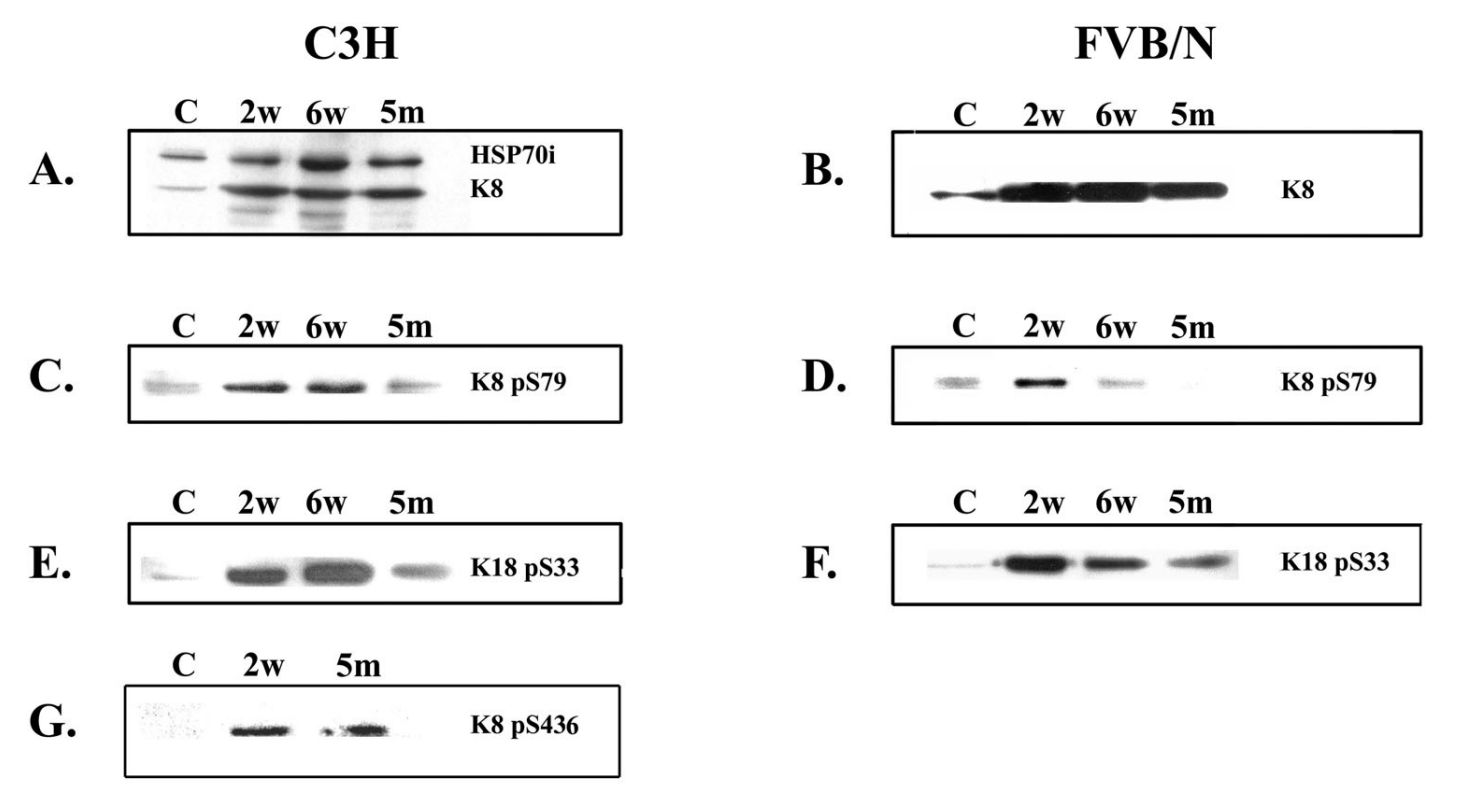
**Biochemical analysis of livers from C3H and FVB/n mice. **Western blots from C3H mouse livers; A, K8 and HSP70i; C, K8 pS79; E, K18 pS33; G, K8 pS436. Western blots from FVB/n mouse livers; B, K8; D, K8 pS79; F, K18 pS33.

The modifications in the amount of HSP70i, K8/18, and phosphorylated keratins (K8 pS79, K8 pS436 and K18 pS33) were analyzed by Western Blotting of total proteins from control and GF-treated C3H and FVB/n mouse livers (2 weeks, 6 weeks and 5 months). GF intoxication induced an increase in keratin levels in livers from both mouse strains (Fig. 1A,1B). HSP70i, which was present in control livers of both mouse strains, was also increased by the treatment (Fig. 1A).

Total proteins from C3H and FVB/n livers were probed with antibodies against K8 pS79, K8 pS436 and K18 pS33 (Fig. 1C,1D,1E,1F,1G). Significant changes in K8 and K18 phosphorylation occurred after GF-treatment in both mouse strains (Fig. 1C,1D,1E,1F,1G). Small amounts of K8 pS79 and K18 pS33 were found in control livers (Fig. 1C,1D and 1E,1F), whereas K8 pS436 was not detected (Fig. 1G). After 2 weeks of treatment, an increase in the amount of all phosphokeratin species studied was observed. The phosphorylation levels of K8 S436 and K18 S33 remained higher than control values in both mouse strains for the entire treatment (Fig. 1E,1F,1G). However, when compared with 2 week treatment, a decrease in K8 pS436 and K18 pS33 was noted after 5 months of treatment (Fig. 1E,1F,1G). Similarly, a decrease in K8 S79 phosphorylation was observed after 5 months of treatment, in C3H mice (Fig. 1C). However in FVB/n mice, K8 pS79 was not detected after the same period of treatment (Fig. 1D).

### Localization of HSP70i and K8/18 during GF intoxication

We analyzed at the cellular level, by double immunofluorescence staining, the distribution of HSP70i and IFs on liver sections of control and GF-treated C3H and FVB/n mouse livers.

**Figure 2:**
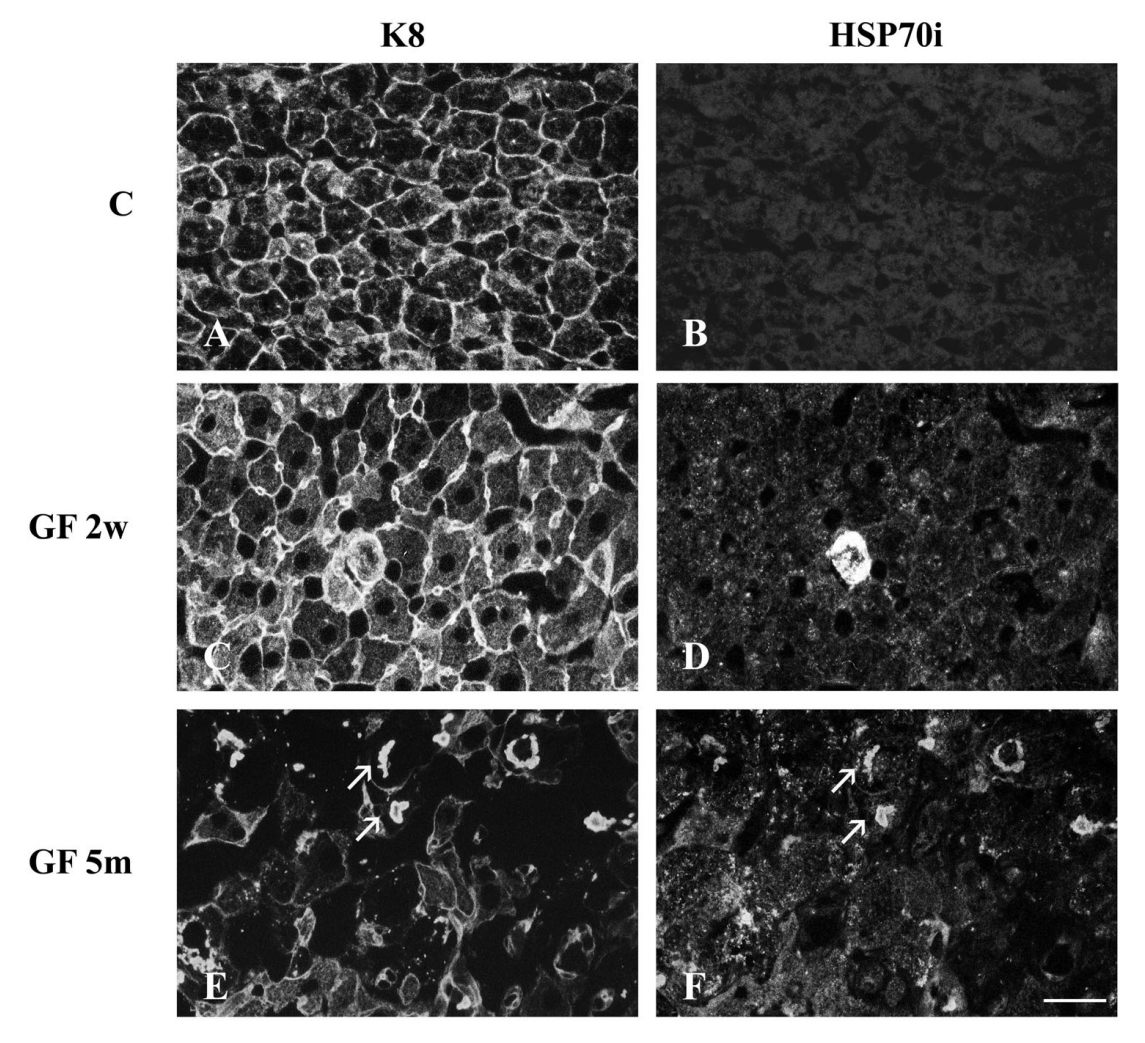
**Distribution of keratin IFs and HSP70i in hepatocytes from control and GF-fed C3H mice. **A, C, E keratin IFs; B, D, F HSP70i; A, B) control; C, D) 2 week treatment; E, F) 5 month treatment. Arrows in E and F indicate reactive MBs with Troma 1 (anti-K8) and anti-HSP70i, respectively. Scale bar = 20 μm.

In control hepatocytes, IFs formed a complex cytoplasmic network that was denser at the cell membrane and particularly around the bile canaliculi (Fig. 2A). Our biochemical analysis showed that HSP70i was present in control hepatocytes. However, by immunofluorescence, we did not detect the presence of HSP70i in the cells (Fig. 2B). After 2 weeks of treatment, most of the hepatocytes were enlarged and the bile canaliculi were dilated. IF network was denser around dilated bile canaliculi (Fig. 2C). All hepatocytes contained a very dense cytoplasmic IF network. These modifications were accompanied by an increase in the amount of HSP70i in hepatocytes and a granular staining was detectable at the membrane and in the nuclei (Fig. 2D). A few cells showed a high level of HSP70i. After 5 months of treatment, there was a mosaic pattern of cells with and without IF staining (Fig. 2E). HSP70i showed a granular staining pattern in many hepatocytes and was also present in MBs (Fig. 2F).

### Phosphorylation of K8 S79, K8 S436 and K18 S33 during GF intoxication

**Figure 3:**
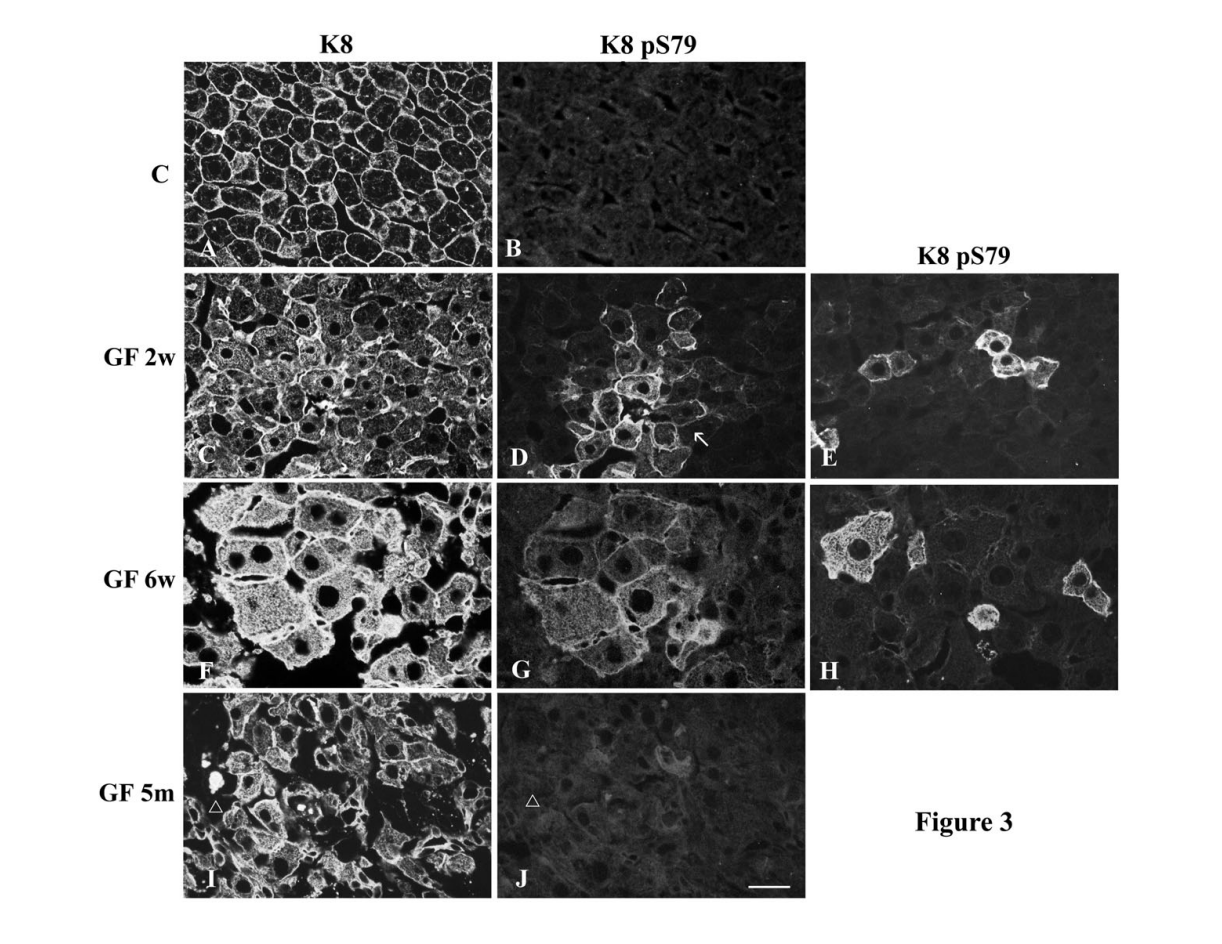
**Distribution of keratin IFs and K8 pS79 in hepatocytes from control and GF-fed C3H mice. **A, C, F, I keratin IFs; B, D, E, G, H, J K8 pS79; A, B) control; C, D, E) 2 week treatment; F, G, H 6 week treatment; I, J 5 month treatment. Arrow in D indicates clusters of cells containing K8 pS79. Empty arrowheads in I and J indicate MBs reactive with Troma 1 but not with LJ4 (anti-K8 pS79), respectively. Scale bar = 20 μm.

**Figure 4:**
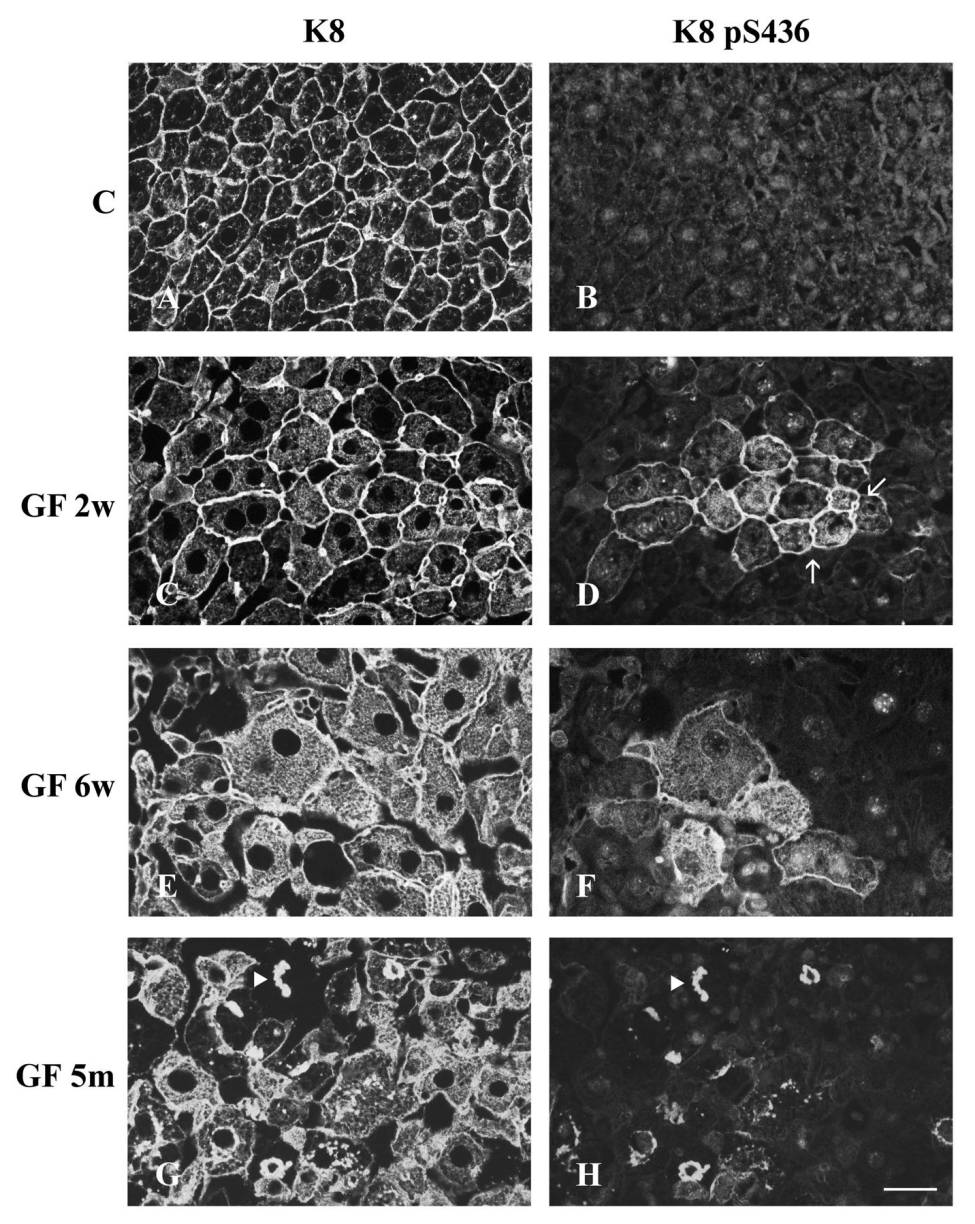
**Distribution of keratin IFs and K8 pS436 in hepatocytes from control and GF-fed C3H mice. **A, C, E, G keratin IFs; B, D, F, H K8 pS436; A, B) control; C, D) 2 week treatment; E, F) 6 week treatment; G, H); 5 month treatment. Arrows in D indicate clusters of cells containing K8 pS436. Filled arrowheads in G and H indicate reactive MBs with Troma 1 and 5B3 (anti-K8 pS436), respectively. Scale bar = 20 μm.

**Figure 5:**
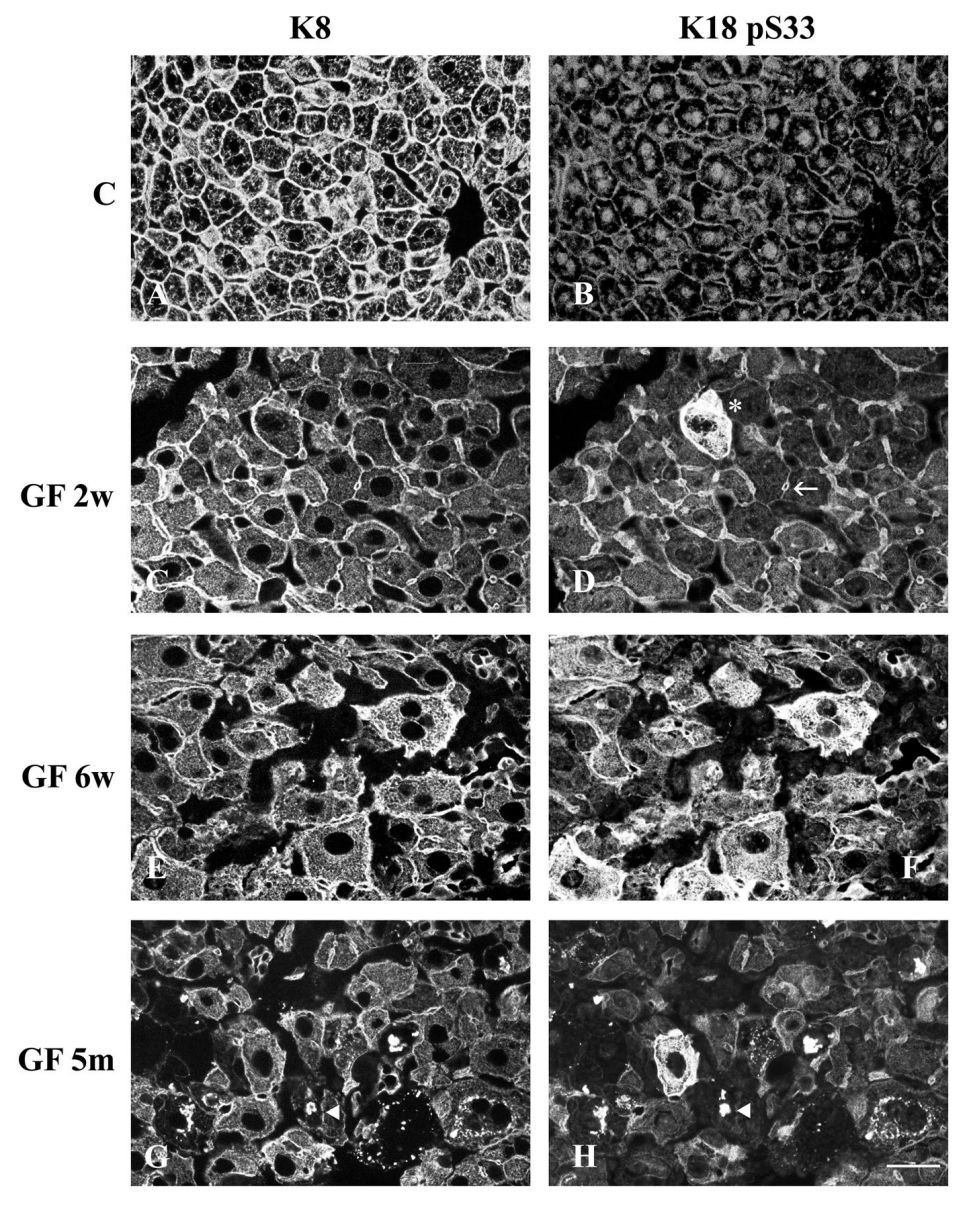
**Distribution of keratin IFs and K18 pS33 in hepatocytes from control and GF-fed C3H mice. **A, C, E, G keratin IFs; B, D, F, H K18 pS33; A, B) control; C, D) 2 week treatment; E, F) 6 week treatment; G, H) 5 month treatment. Asterisk in D shows an hepatocyte containing a high level of K18 pS33; arrow indicates a dilated bile canaliculi. Filled arrowheads in G and H indicate reactive MBs with Troma 1 and Ab8250 (anti-K18 pS33), respectively. Scale bar = 20 μm.

Cryosections of control and GF-treated C3H and FVB/n mouse livers were fixed with 4% paraformaldehyde and processed for double immunofluorescence staining. As mentioned above, control mice showed hepatocytes with a cytoplasmic IF network, which was denser at the cell periphery (Fig. 3,4,5A). K8 pS79 and K8 pS436 were not generally detected in the IF network of control hepatocytes. Only, occasionally some doublet cells, most likely representing cells in mitosis, were stained (data not shown). A basal level of phosphorylation for K18 S33 was detected at the periphery of all hepatocytes (Fig. 5B).

**Figure 6:**
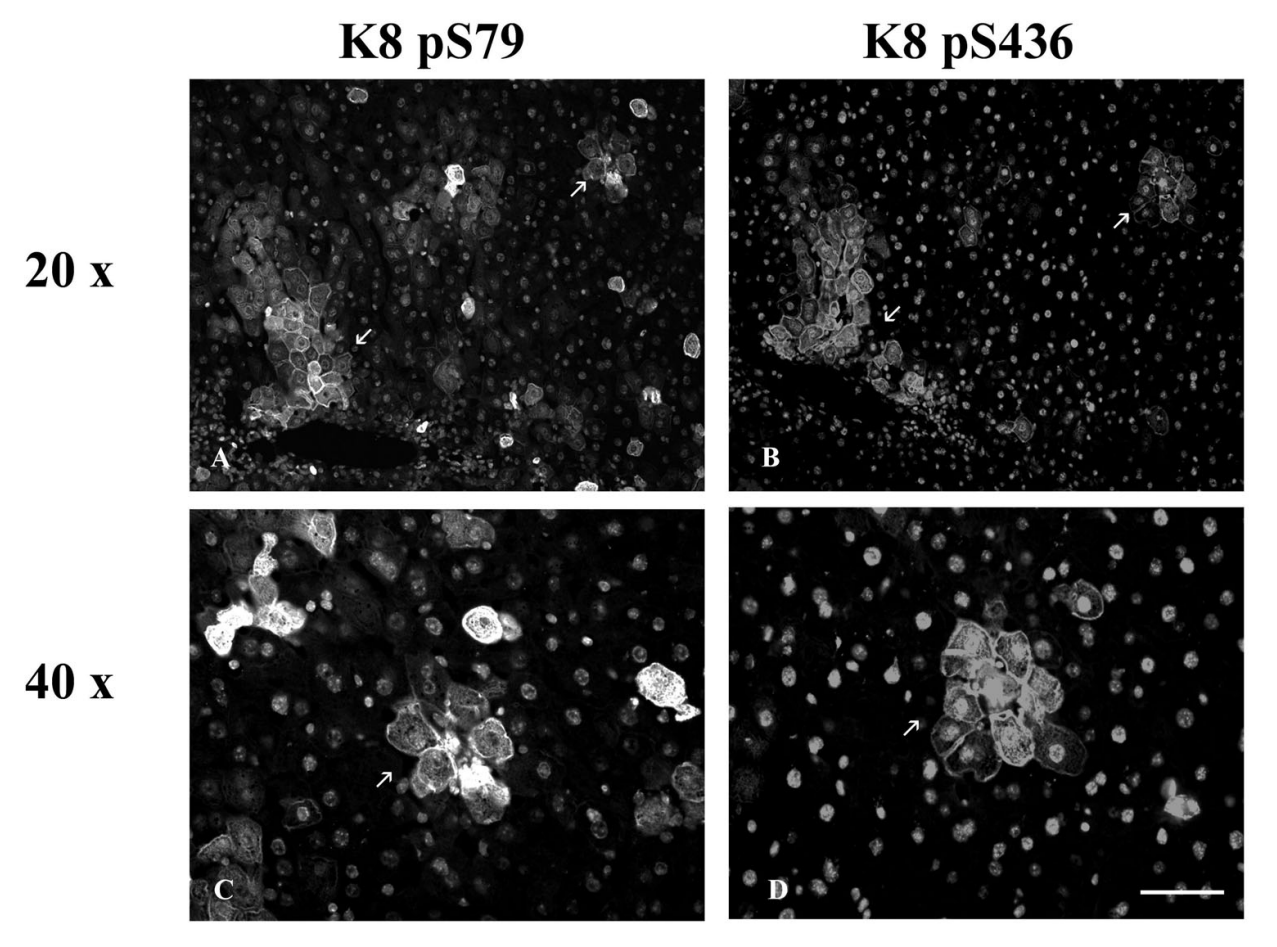
**Colocalization of K8 pS79 and K8 pS436 in hepatocytes from GF-fed C3H dmice. **A, C K8 pS79; B, D K8 pS436; A, B, C, D 2 week treatment. Arrows in A and B indicate clusters of hepatocytes containing both K8 pS79 and K8 pS436. Note: reactivity in nuclei observed in A, B, C and D represents non-specific staining due to the secondary antibody. Scale bar = 20 μm.

After 2 weeks of GF-treatment, hepatocytes were enlarged and an increase in cytoplasmic IF network was observed (Fig. 3,4,5C). This treatment induced the phosphorylation of K8 on S79 and S436 in some hepatocytes. K8 pS79 and K8 pS436 were present in clusters of cells scattered over the whole liver (Fig. 3,4,7D). The groups of cells stained with the anti-K8 pS79 or anti-K8 pS436 usually surrounded damaged cells (Fig. 3,7D). In the case of K8 pS79, IFs located in the cytoplasm and at the periphery of the cells were highly stained (Fig. 3D,3E). For K8 pS436, the staining was stronger at the cell periphery and around the dilated bile canaliculi (Fig. 4D). In addition to their presence in clusters of cells, K8 pS79 and K8 pS436 displayed an intense cytoplasmic staining in some isolated cells or cell doublets (Fig. 3E). Since both epitopes showed similarities in their patterns of distribution, we asked whether they were present in the same hepatocytes. Immunostaining for the detection of K8 pS79 and K8 pS436 were performed on serial liver sections of GF-treated mouse liver. Our results showed that the groups of hepatocytes positives for K8 pS79 were also positive for K8 pS436 (Fig. 6).

**Figure 7:**
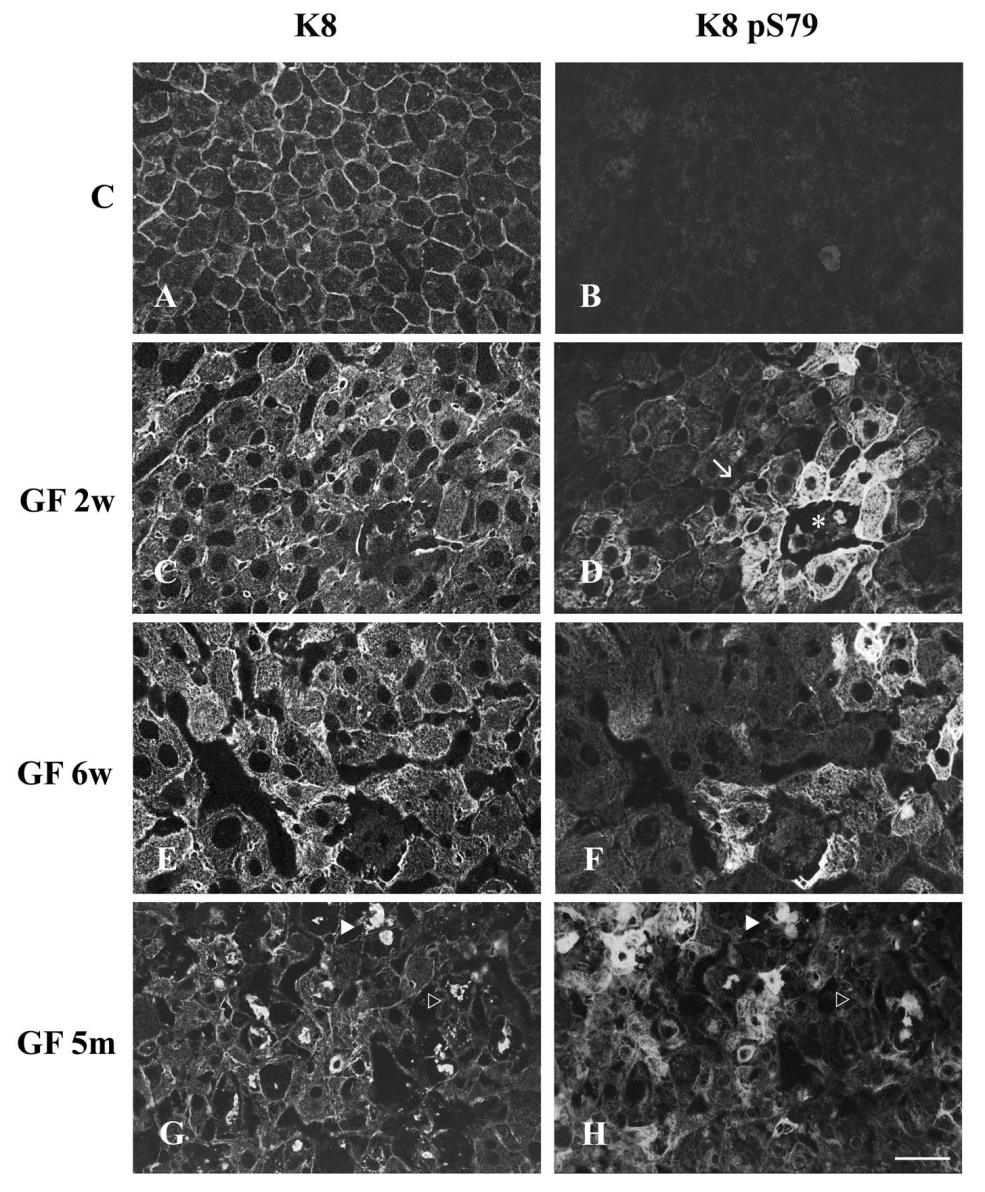
**Distribution of keratin IFs and K8 pS79 in hepatocytes from control and GF-fed C3H mice. **A, C, E, G keratin IFs; B, D, F, H K8 pS79; A, B) control; C, D) 2 week treatment; E, F) 6 week treatment; G, H) 5 month treatment. Arrow in D indicates clusters of cells containing K8 pS79; asterisk shows a damaged hepatocyte. Filled arrowheads in G and H indicate MBs reactive with Troma 1 and LJ4 (anti-K8 pS79), respectively; empty arrowheads indicate MBs reactive with Troma 1 but not with LJ4 (anti-K8 pS79), respectively. Scale bar = 20 μm.

In the case of K18 S33, its phosphorylation was increased in most (if not all) hepatocytes. Most of the staining was observed at the periphery of the cells, delimitating clearly the bile canaliculi. A few hepatocytes showed high levels of cytoplasmic K18 pS33 (Fig. 5D).

After 6 weeks of GF-treatment, the distribution of hepatocytes containing K8 pS79 and K8 pS436 was different from the one observed after 2 weeks of treatment. Clusters of labeled cells were smaller, whereas labeled isolated cells became more prominent (Fig. 3G,4F). Singlet and doublet cell(s) highly labeled with K8 pS79 and K8 pS436 were also present (Fig. 3H). K18 pS33 was present in most hepatocytes and showed a similar pattern as the one observed after staining with Troma1 (Fig. 5F).

**Figure 8:**
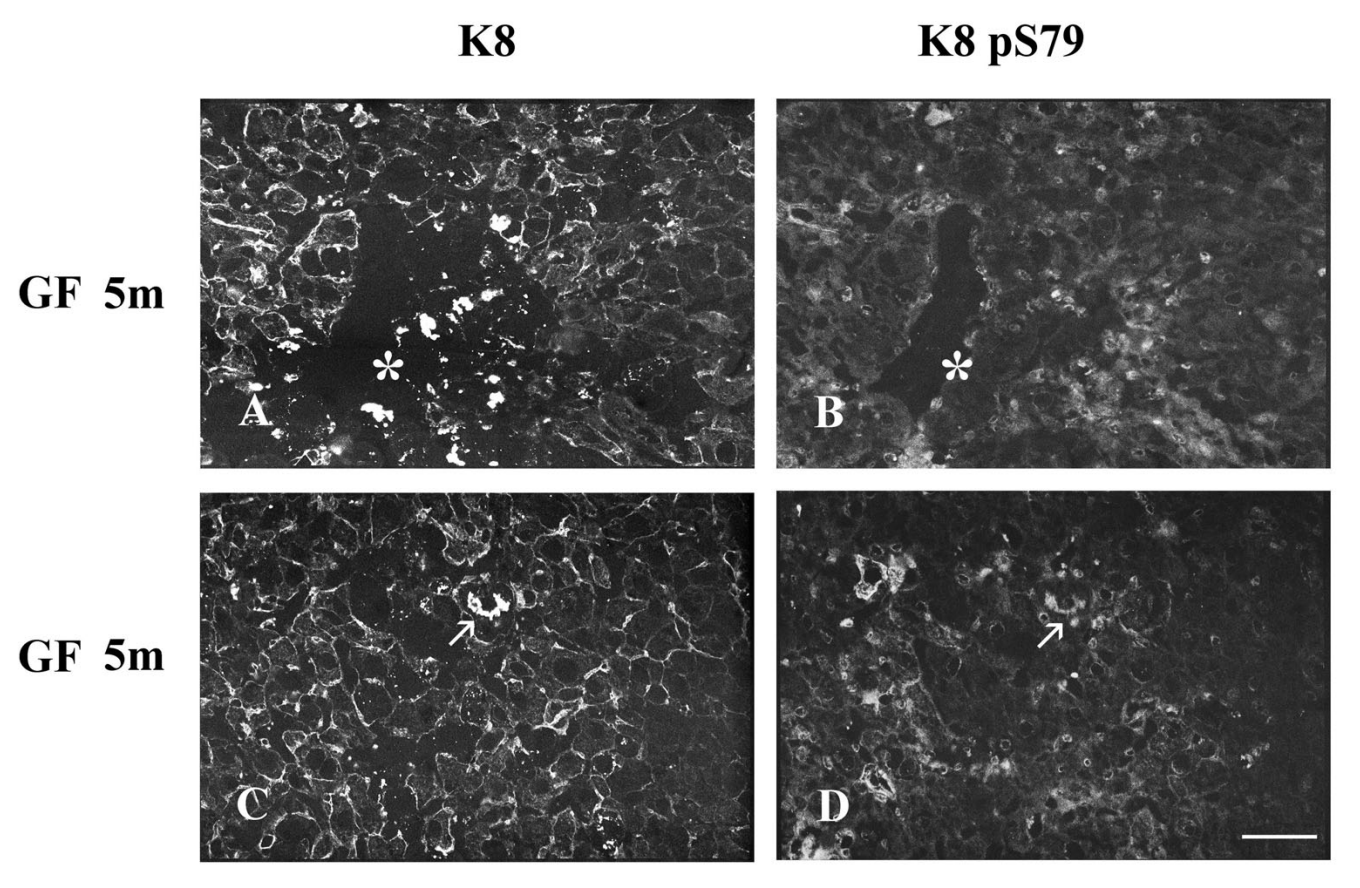
**Distribution of keratin IFs and K8 pS79 in hepatocytes from GF-fed FVB/n mice. **A, C keratin IFs; B, D K8 pS79; A, B, C, D 5 month treatment. Asterisks in A and B indicate MBs reactive with Troma 1 but not with LJ4; arrows in C and D indicate MBs reactive with Troma 1 and LJ4, respectively. Scale bar = 20 μm.

After 5 months of GF-treatment, MBs were present in some hepatocytes in both mouse strains (Fig. 3I,4G,5G). MBs had variable size and different positions depending on the cell and were observed in cells with or without a visible intracytoplasmic IF network, as detected with Troma 1. In both mouse strains, K8 pS436 and K18 pS33 were present in MBs (Fig. 4,5H), whereas K8 pS79 seemed to be absent (Fig. 3J). Experiments described above were also performed using cold acetone instead of 4% paraformaldehyde. After acetone fixation, no difference in the staining pattern was observed for GF-treatment of 2 and 6 weeks in both mouse strains (Fig. 7). However, differences were observed for MB staining in the 5 months GF-treated mouse liver. In C3H mouse strain livers, K8 pS79 was present in many MBs although some of them showed no staining (Fig. 7H). In FVB/n mouse strain livers, K8 pS79 was not present in most MBs (Fig. 8). No difference in staining of MBs was observed for K8 pS436 and K18 pS33.

### Localization of phosphorylated K8 species and HSP70i during GF intoxication

**Figure 9:**
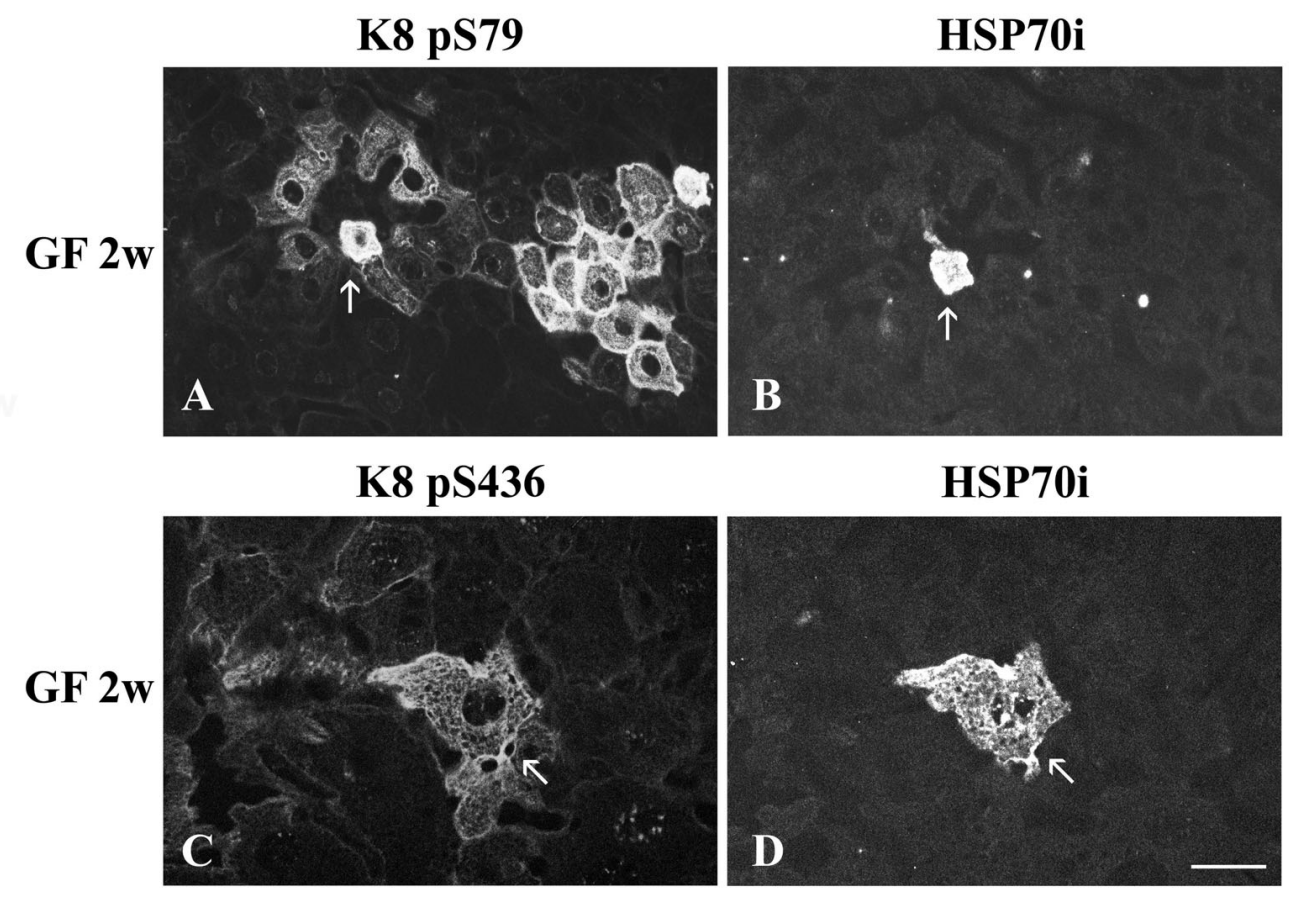
**Distribution of phosphorylated keratin IFs and HSP70i in hepatocytes from GF-fed C3H mice. **A K8 pS79; B, D HSP70i; C K8 pS436; A, B, C, D 2 week treatment. Arrows in A and B indicate cells in which HSP70i and K8 pS79 colocalized. Arrows in C and D indicate cells in which HSP70i and K8 pS436 colocalized. Scale bar = 20 μm.

Double immunostaining with anti-HSP70i and anti-phosphorylated keratins (K8 pS79 or K8 pS436) was performed for studying the localization of HSP70i in relation to keratin phosphorylation. The results showed that HSP70i and phosphorylated K8 species colocalized in some cells (Fig. 9). However, in most of the cells, the colocalization was not observed.

## Discussion

The functional significance of K8/18 in simple epithelium has been the subject of numerous studies over the last decade [29-35]. Although most of these reports lead towards roles for K8/18 in the resistance of cells to mechanical and toxic stress, the molecular mechanisms underlying these phenomena remain to be elucidated. To date, most of our understanding of the pathways involving keratins in the response of hepatocytes to toxic stress comes from the analyses of various cell lines [36-38]. K8/18 phosphorylation at specific sites has been proposed to be a key factor in the regulation of those keratin functions. In this regard, K8 pS79, K8 pS436, K18 pS52 and K18 pS33 are the most studied phosphorylation sites [39].

*In vivo*, K8/18 are also subjected to phosphorylation and, as suggested by *in vitro *studies, it is proposed to help hepatocytes to cope with toxic stress [26,29,35]. For instance, transgenic mice expressing human K18 S52 mutated in alanine mutant are more susceptible to drug-induced liver injury than transgenic mice over expressing wild type human K18 [40].

In the present study, we showed that the chronic intoxication of mice with GF, which is known to induce modifications in keratin organization and formation of MBs [33], was associated with increased expression of the stress protein HSP70i. GF-treatment resulted in a rapid increase in the expression of HSP70i. This modification was already perceptible after 2 weeks of treatment and was maintained for the whole period of treatment. This result provides direct evidence that GF-treatment, which has been proposed to constitute an oxidative stress for hepatocytes [41], triggers signaling pathways involved in cellular protection [33]. This interpretation of our biochemical data is in agreement with our immunofluorescence study, which showed that HSP70i partly relocalized to the nucleus during the treatment. This distribution pattern is typical of the distribution of HSP70i in stressed cells [42,43].

We have previously shown that GF intoxication induced an overall increase in K8/18 phosphorylation [44,45]. Here, we show that GF-treatment is associated with modifications in K8/K18 phosphorylation at specific sites such as: K8 S79, K8 S436 and K18 S33. Among the studied phosphorylation sites, and because it was present and was increased in all treated hepatocytes, K18 pS33 was the keratin phosphoepitope the tissue distribution of which resembled that of HSP70i. The phosphorylation of K18 S33 has been shown to play a role in keratin reorganization during mitosis and by linking 14-3-3 proteins, to modulate their function [46,47]. Hence, we propose that K18 S33 phosphorylation could be linked to IF reorganization during GF intoxication. Moreover, because K18 pS33 is increased in all hepatocytes, it could be implicated in the stress response by participating in the relocalization and/or the recruitment of molecules or factors implicated in stress-induced cell signaling.

In contrast to K18 pS33, phosphorylated K8 species, K8 pS79 and K8 pS436 were not present in control mice hepatocytes. After 2 and 6 weeks of treatment, we observed an increase in the level of phosphorylation on these sites. However, contrary to HSP70i and K18 pS33, these phosphorylation sites were only present in isolated cells (singlet or doublets) or clusters of cells. Labeled singlet or doublet cells were more numerous after staining with the anti-K8 pS79 than after staining with the anti-K8 pS436. These cells could correspond to cells that are undergoing mitosis. This is supported by previous studies which showed that the phosphorylation of K8 on S79 and S436 occurs during mitosis [25,48]. This interpretation is also in agreement with the work of Stumptner et al. [49], which showed the presence of cell doublets reactive with the anti-K8 pS79 after a short treatment with DDC that induces on the long term MB formation. The discrepancy in the number of cells stained for K8 pS79 and K8 pS436, both in the singlet and doublet cells, suggests that different kinases are involved in the phosphorylation of those sites.

The presence of K8 pS79 and K8 pS436 was also detected in islets of cells. Interestingly, both antigens were present in the same clusters of cells surrounding unstained cells that were most likely undergoing apoptosis. These unstained cells are evocative of detached cells during anoikis, an apoptotic process that can be induced by loss of cell-cell anchorage. Stress and apoptosis has been shown to modulate K8 S79 and K8 S436 phosphorylation [25,48]. The observed phosphorylation could indicate that these hepatocytes are stressed hepatocytes intended to apoptosis. However, analysis of the livers for the presence of apoptosis showed that only a few hepatocytes are going through programmed cell death and groups of cells in apoptosis were never observed (data not shown). We propose that the apoptotic cell could represent the starting point of a signal transduction pathway to neighboring cells. The activation of specific kinases that would phosphorylate keratins could provide those cells a resistance to apoptosis. This latter interpretation is in agreement with the notion that K8/18 intermediate filaments play a key role in the protection of cells against apoptosis [26,35].

Liao et al. [50] have shown that HSP70 associates with K8/18 *via *K8. Our study show that colocalization of HSP70i and IFs occurs only in a few hepatocytes. Since the hepatocytes, in which colocalization was observed, contained K8 pS79 or K8 pS436, HSP70i binding to IFs in these cells may be related to the presence of keratin phosphorylation and participates to cellular pathways involving phosphorylated K8/18 on specific sites. Ku et al. [51] have shown that phosphorylation could modulate K8/18 ubiquitination and ensuing turnover. Knowing that binding of HSP70 to a protein can affect its targeting by kinases or phosphatases [52], HSP70i could bind to phosphorylated K8 species, prevent dephosphorylation by specific phosphatases, and thereby enhance phosphorylation-mediated K8/18 protection from degradation by the ubiquitin pathway [51,53]. However, since HSP70i and phosphorylated K8 species colocalized only in a few cells over the whole tissue, the relevance of this phenomenon in the response to the presence of the hepatotoxin needs to be addressed and further investigations will be necessary to confirm that hypothesis.

Chronic intoxication of mice with GF induces the formation of MBs. Numerous studies have demonstrated the presence of different phosphorylated K8/18 species within MBs, suggesting that K8/18 phosphorylation could participate in the MB formation processes [49,54]. In our experiments, we showed that K8 pS436 and K18 pS33 were present in all observed MBs, whereas K8 pS79 was present in MBs in C3H mice hepatocytes but not in FVB/n mice. The difference in the presence of K8 pS79 phosphoepitope within MBs suggests that phosphorylation at that specific site is not essential for MB formation. However, as suggested by Stumptner et al. [49], because K8 pS436 and K18 pS33 are always detected in MBs, phosphorylation on these sites could be implicated in the processes of MB formation. Taken together, those results indicate that in the context of MB formation, K8/18 phosphorylation should not be considered as a general phenomenon but as specific events that affect precise sites on K8 or K18. The difference observed between keratin phosphorylation in C3H and FVB/n mice indicates that the genetic background influences the response of hepatocytes to toxic stress. This interpretation is in agreement with the results obtained with K8-null mice which displayed variable phenotypes depending on the genetic background [30,31].

The treatment with GF that represents a toxic stress, most likely, involves the activation of stress activated protein kinases (SAPKs) in some hepatocytes. SAPKs p38 and JNK are physiologic kinases for K8 S79 and K8 S436 [37,55]. We postulate that p38 kinase and/or JNK are activated by GF-treatment in some hepatocytes and are responsible for the modifications in K8 phosphorylation we observed. K8 and K18 give different patterns of phosphorylated cells indicating that, under the same conditions, K8 and K18 phosphorylation is regulated differently.

## Conclusions

Our results show that increases in HSP70i, K8/18 expression and K8/18 phosphorylation constitute early events in the response of hepatocytes to the presence of GF. These observations support a role for keratins in preserving cellular integrity during stress conditions induced by the presence of a chemical agent [33,35]. HSP70i expression in hepatocytes after GF-treatment is not directly related to K8/18 phosphorylation at the studied sites: K8 S79, K8 S436 and K18 S33. With regard to MB formation, it appears that both HSP70i and K8/18 phosphorylation might contribute to the IF aggregation processes. The involvement of K8/18 phosphorylation in MB formation seems to be related only to specific sites and dependent on mouse genetic inheritance.

## Methods

### Experimental design

Experiments were performed with adult C3H mice (Charles River Canada, St-Constant, QC) and FVB/n mice (Baribault et al. 1994) weighing 25 to 30 g. Two mouse strains were used to minimize the potential effect of different genetic background on the response of hepatocytes and to facilitate the interpretation of the data. All animals were housed with a 12-hour light-dark cycle and allowed the consumption of water and of a standard mouse semi-synthetic diet (Texlad Test Diet, Madison, WI), both *ad libitum*. GF-treated mice were fed a diet containing 2.5% (w/w) GF (Schering Corp., Kenilworth, NJ) for different periods of time: 2 weeks, 6 weeks and 5 months according to the method of Denk et al. [28]. Control mice were fed the same diet without GF. For control and each period of GF-treatment, experimental groups included 3 animals. Mice were sacrificed by cervical dislocation and livers were snap frozen in methylbutane precooled with liquid nitrogen and stored at -70°C before use. All experiments were conducted according to the requirements of Canadian Council Animal Care and the "Université du Québec à Trois-Rivières" Animal Welfare Committee. For microscopical studies, paraformaldehyde and cold acetone were routinely used, as fixatives, to ensure that the staining patterns were not a consequence of the fixative used.

### Reagents

The antibodies used were as following: Troma 1, a rat monoclonal antibody (rAb) that recognizes K8 [56]; LJ4, a mouse monoclonal antibody (mAb) that recognizes human K8 pS73 equivalent to mouse K8 pS79 [25]; mAb 5B3 that recognizes K8 pS431 equivalent to mouse K8 pS436 [48]; 8250, a rabbit polyclonal antibody (pAb) that recognizes K18 pS33 [46] and a pAb that recognizes the stress inducible form of HSP70, HSP70i (Stressgen, Victoria, BC). The secondary antibodies for fluorescence microscopy were as follows: tetramethylisothiocyanate (TRITC) or fluorescein isothiocyanate (FITC) conjugated goat anti-rat IgG, FITC-conjugated donkey anti-rabbit IgG (Jackson Immunoresearch, Bio/Can Scientific, Mississauga, ON). The M.O.M. kit and Avidin/Biotin blocking kit (Vector^® ^Laboratories Canada, Burlington, ON) were used to perform immunolabelling with mAbs LJ4 and 5B3. The secondary antibodies used for Western blotting were as follows: biotinylated goat anti-rat IgG, biotinylated donkey anti-mouse IgG and peroxydase donkey anti-rabbit IgG (Jackson Immunoresearch, Bio/Can Scientific, Mississauga, ON). Other reagents used were: Horseradish Streptavidin Peroxydase-conjugated (SPC) (Jackson Immunoresearch, Bio/Can Scientific, Mississauga, ON), Bovine Serum Albumin (BSA) (Jackson Immunoresearch, Bio/Can Scientific, Mississauga, ON), Leupeptin (Sigma-Aldrich Canada, Oakville, ON), Pepstatin (Sigma-Aldrich Canada, Oakville, ON), Aprotinin (Sigma-Aldrich Canada, Oakville, ON), Normal Horse Serum (NHS) (Vector^® ^Laboratories Canada, Burlington, ON), Luminol (Amersham Pharmacia Biotech, Oakville, ON).

### Gel electrophoresis and immunoblotting

Livers were homogenized in 62.5 mM Tris-HCl, pH 6.8 containing 2.3 % (w/v) SDS, 50 mM sodium fluoride, 10 mM EDTA, 1 mM sodium pyrophosphate, 1 mM DTT, 1 mM PMSF, 1 μM leupeptin (Sigma-Aldrich Canada, Oakville, ON), 1 μM pepstatin (Sigma-Aldrich Canada, Oakville, ON), 2.5 μg/ml aprotinin (Sigma-Aldrich Canada, Oakville, ON). Proteins were separated by electrophoresis on 10% SDS-polyacrylamide gels [57]. Protein concentration was determined by the Lowry method, modified for the presence of SDS [58], and equal amounts of proteins (5 to 12.5 μg) were loaded on each well. Gels were stained with 0.1% Coomassie Blue, or transferred onto nitrocellulose membranes (Biorad laboratories Canada, Mississauga, ON), and processed for immunodetection. Membranes were blocked overnight with 5% (w/v) non-fat dry milk (Carnation, Nestlé^®^) in PBS (Phosphate Buffer Saline, 0.137 M NaCl, 2.7 mM KCl, 4.3 mM Na_2_HPO_4_, 14.7 mM KH_2_PO_4,_pH 7.2), incubated with the primary antibodies for 45 min, at room temperature, washed in PBS containing 0.2% (v/v) Tween 20 and incubated for 45 min with the appropriate secondary antibody: biotinylated goat anti-rat IgG (Jackson Immunoresearch, Bio/Can Scientific, Mississauga, ON), biotinylated donkey anti-mouse IgG (Jackson Immunoresearch, Bio/Can Scientific, Mississauga, ON) and horseradish peroxydase donkey anti-rabbit IgG (Jackson Immunoresearch, Bio/Can Scientific, Mississauga, ON). When biotinylated secondary antibodies were used, membranes were washed with PBS-Tween 20 and incubated with streptavidin conjugated with horseradish peroxydase (Jackson Immunoresearch, Bio/Can Scientific, Mississauga, ON) for 30 min and washed with PBS-Tween 20. The chemiluminescent horseradish peroxydase substrate Luminol (Amersham Pharmacia Biotech, Oakville, ON) was added to the membranes according to recommendations of the company, and membranes were exposed to Blue X-Omat X-ray film sheets (Mandel Scientific Company, Guelph, ON) to localize antibody binding.

### Fluorescence microscopy

Cryosections (4 μm) of fresh liver were fixed with 4% (w/v) paraformaldehyde in PBS pH 7.2 for 20 min, at room temperature, and rinsed in PBS or TBS (Tris Buffer Saline, 10 mM Tris-HCl, 0.138 M NaCl, 2.7 mM KCl, pH 7.4) upon staining protocols. Since fixation can affect antibody-binding capacity, cryosections were also fixed with cold acetone (-20°C) for 10 min. For the detection of K8, sections were incubated with rAb Troma 1 at room temperature, washed in PBS and incubated with a FITC or a TRITC conjugated goat anti-rat IgG (Jackson Immunoresearch, Bio/Can Scientific, Mississauga, ON) for 45 min, at room temperature. For immunostaining of K18 pS33, sections were incubated for 1 hour, at room temperature with anti-K18 pS33 (8250) diluted in PBS containing 10% (w/v) BSA, washed in PBS and incubated for 45 min with a FITC conjugated donkey anti-rabbit IgG in PBS containing 10% BSA (Jackson Immunoresearch, Bio/Can Scientific, Mississauga, ON). Immunostaining with anti-K8 pS79 (LJ4) and anti-K8 pS436 (5B3) mAbs, was done using the M.O.M. (mouse on mouse) detection kit (Vector^® ^Laboratories Canada, Burlington, ON) and an Avidin/Biotin blocking kit (Vector^® ^Laboratories Canada, Burlington, ON) according to recommendations of the company. Normal horse serum (Vector^® ^Laboratories Canada, Burlington, ON) was added to solution during incubation step with secondary antibody. For heat shock proteins staining, liver sections were incubated with anti-HSP 70i diluted in TBS containing 10% BSA for 45 min at 37°C, washed in TBS and incubated for 45 min at 37°C with a FITC conjugated donkey anti-rabbit IgG (Jackson Immunoresearch, Bio/Can Scientific, Mississauga, ON) diluted in TBS containing 10% BSA. For detection of HSP70i, K8 pS79 and K8 pS436, the sections were treated with 1% (v/v) Nonidet P-40 (Sigma-Aldrich Canada, Oakville, ON) following fixing step with 4% paraformaldehyde.

The tissues were mounted in P-phenylene diamine diluted in 50% (v/v) glycerol. The slides were kept at -20°C and photomicrographs were collected using an Olympus^® ^BX60 photomicroscope.

## List of abbreviations

HSP70i – inducible form of 70 kDa Heat shock protein. GF – griseofulvin. IFs – intermediate filaments. K8 – keratin 8. K8/18 – keratin 8 and keratin 18. K8 S79 – serine 79 on keratin 8. K8 pS79 – phosphorylated serine 79 on keratin 8. MBs – Mallory bodies.

## Authors' contributions

MF carried out all western blotting analyses, performed the immunofluorescence studies and participated in drafting the manuscript. LV participated in the design of the study. MC participated in the design of study, its coordination and drafting the manuscript. All authors read and approved the final manuscript.

## References

[B1] Lazarides E (1980). Intermediate filaments as mechanical integrators of cellular space. Nature.

[B2] Fuchs E, Weber K (1994). Intermediate filaments: structure, dynamics, function, and disease. Annu Rev Biochem.

[B3] Schliwa M, Springer-Verlag (1986). The cytoskeleton: An introductory survey. In : Alfert M, Beerman W, Goldstein L, Porter K R, eds Cell Biology Monograhs.

[B4] Herrmann H, Hesse M, Reichenzeller M, Aebi U, Magin TM (2003). Functional complexity of intermediate filament cytoskeletons: from structure to assembly to gene ablation. Int Rev Cytol.

[B5] Steinert PM, Roop DR (1988). Molecular and cellular biology of intermediate filaments. Annu Rev Biochem.

[B6] Moll R, Franke WW, Schiller DL, Geiger B, Krepler R (1982). The catalog of human cytokeratins: patterns of expression in normal epithelia, tumors and cultured cells. Cell.

[B7] Vassar R, Coulombe PA, Degenstein L, Albers K, Fuchs E (1991). Mutant keratin expression in transgenic mice causes marked abnormalities resembling a human genetic skin disease. Cell.

[B8] Fuchs E, Cleveland DW (1998). A structural scaffolding of intermediate filaments in health and disease. Science.

[B9] Coulombe PA, Omary MB (2002). 'Hard' and 'soft' principles defining the structure, function and regulation of keratin intermediate filaments. Curr Opin Cell Biol.

[B10] Coulombe PA, Hutton ME, Letai A, Hebert A, Paller AS, Fuchs E (1991). Point mutations in human keratin 14 genes of epidermolysis bullosa simplex patients: genetic and functional analyses. Cell.

[B11] Porter RM, Lane EB (2003). Phenotypes, genotypes and their contribution to understanding keratin function. Trends Genet.

[B12] Loranger A, Duclos S, Grenier A, Price J, Wilson-Heiner M, Baribault H, Marceau N (1997). Simple epithelium keratins are required for maintenance of hepatocyte integrity. Am J Pathol.

[B13] Ku NO, Michie S, Oshima RG, Omary MB (1995). Chronic hepatitis, hepatocyte fragility, and increased soluble phosphoglycokeratins in transgenic mice expressing a keratin 18 conserved arginine mutant. J Cell Biol.

[B14] Ku NO, Darling JM, Krams SM, Esquivel CO, Keeffe EB, Sibley RK, Lee YM, Wright TL, Omary MB (2003). Keratin 8 and 18 mutations are risk factors for developing liver disease of multiple etiologies. Proc Natl Acad Sci U S A.

[B15] Ku NO, Gish R, Wright TL, Omary MB (2001). Keratin 8 mutations in patients with cryptogenic liver disease. N Engl J Med.

[B16] Ku NO, Wright TL, Terrault NA, Gish R, Omary MB (1997). Mutation of human keratin 18 in association with cryptogenic cirrhosis. J Clin Invest.

[B17] Denk H, Krepler R, Lackinger E, Artlieb U, Franke WW (1982). Immunological and biochemical characterization of the keratin-related component of Mallory bodies: a pathological pattern of hepatocytic cytokeratins. Liver.

[B18] Hazan R, Denk H, Franke WW, Lackinger E, Schiller DL (1986). Change of cytokeratin organization during development of Mallory bodies as revealed by a monoclonal antibody. Lab Invest.

[B19] Jensen K, Gluud C (1994). The Mallory body: theories on development and pathological significance (Part 2 of a literature survey). Hepatology.

[B20] Jensen K, Gluud C (1994). The Mallory body: morphological, clinical and experimental studies (Part 1 of a literature survey). Hepatology.

[B21] Denk H, Stumptner C, Zatloukal K (2000). Mallory bodies revisited. J Hepatol.

[B22] Mayer R, Lowe J, Landon M, McDermott H, Tuckwell J, Doherty F, Laszlo L, Springer-Verlag (1991). Ubiquitin and the lysosome system: molecular pathological and experimental findings.. Eds Maresca, B and Lindquist, S.

[B23] Yuan QX, Marceau N, French BA, Fu P, French SW (1995). Heat shock in vivo induces Mallory body formation in drug primed mouse liver. Exp Mol Pathol.

[B24] Riley NE, Li J, McPhaul LW, Bardag-Gorce F, Lue YH, French SW (2003). Heat shock proteins are present in mallory bodies (cytokeratin aggresomes) in human liver biopsy specimens. Exp Mol Pathol.

[B25] Liao J, Ku NO, Omary MB (1997). Stress, apoptosis, and mitosis induce phosphorylation of human keratin 8 at Ser-73 in tissues and cultured cells. J Biol Chem.

[B26] Omary MB, Ku NO, Liao J, Price D (1998). Keratin modifications and solubility properties in epithelial cells and in vitro. Subcell Biochem.

[B27] Toivola DM, Zhou Q, English LS, Omary MB (2002). Type II keratins are phosphorylated on a unique motif during stress and mitosis in tissues and cultured cells. Mol Biol Cell.

[B28] Denk H, Gschnait F, Wolff K (1975). Hepatocellar hyalin (Mallory bodies) in long term griseofulvin-treated mice: a new experimental model for the study of hyalin formation. Lab Invest.

[B29] Ku NO, Zhou X, Toivola DM, Omary MB (1999). The cytoskeleton of digestive epithelia in health and disease. Am J Physiol.

[B30] Baribault H, Penner J, Iozzo RV, Wilson-Heiner M (1994). Colorectal hyperplasia and inflammation in keratin 8-deficient FVB/N mice. Genes Dev.

[B31] Baribault H, Price J, Miyai K, Oshima RG (1993). Mid-gestational lethality in mice lacking keratin 8. Genes Dev.

[B32] Gilbert S, Loranger A, Daigle N, Marceau N (2001). Simple epithelium keratins 8 and 18 provide resistance to Fas-mediated apoptosis. The protection occurs through a receptor-targeting modulation. J Cell Biol.

[B33] Cadrin M, Hovington H, Marceau N, McFarlane-Anderson N (2000). Early perturbations in keratin and actin gene expression and fibrillar organisation in griseofulvin-fed mouse liver. J Hepatol.

[B34] Marceau N, Loranger A, Gilbert S, Daigle N, Champetier S (2001). Keratin-mediated resistance to stress and apoptosis in simple epithelial cells in relation to health and disease. Biochem Cell Biol.

[B35] Omary MB, Ku NO, Toivola DM (2002). Keratins: guardians of the liver. Hepatology.

[B36] Ku NO, Omary MB (1994). Expression, glycosylation, and phosphorylation of human keratins 8 and 18 in insect cells. Exp Cell Res.

[B37] Ku NO, Azhar S, Omary MB (2002). Keratin 8 phosphorylation by p38 kinase regulates cellular keratin filament reorganization: modulation by a keratin 1-like disease causing mutation. J Biol Chem.

[B38] Liao J, Lowthert LA, Omary MB (1995). Heat stress or rotavirus infection of human epithelial cells generates a distinct hyperphosphorylated form of keratin 8. Exp Cell Res.

[B39] Ku NO, Liao J, Chou CF, Omary MB (1996). Implications of intermediate filament protein phosphorylation. Cancer Metastasis Rev.

[B40] Ku NO, Michie SA, Soetikno RM, Resurreccion EZ, Broome RL, Omary MB (1998). Mutation of a major keratin phosphorylation site predisposes to hepatotoxic injury in transgenic mice. J Cell Biol.

[B41] Knasmuller S, Parzefall W, Helma C, Kassie F, Ecker S, Schulte-Hermann R (1997). Toxic effects of griseofulvin: disease models, mechanisms, and risk assessment. Crit Rev Toxicol.

[B42] Stege GJ, Li L, Kampinga HH, Konings AW, Li GC (1994). Importance of the ATP-binding domain and nucleolar localization domain of HSP72 in the protection of nuclear proteins against heat-induced aggregation. Exp Cell Res.

[B43] Welch WJ, Feramisco JR (1984). Nuclear and nucleolar localization of the 72,000-dalton heat shock protein in heat-shocked mammalian cells. J Biol Chem.

[B44] Kawahara H, Cadrin M, French SW (1990). Ethanol-induced phosphorylation of cytokeratin in cultured hepatocytes. Life Sci.

[B45] Cadrin M, Anderson NM, Aasheim LH, Kawahara H, Franks DJ, French SW (1995). Modifications in cytokeratin and actin in cultured liver cells derived from griseofulvin-fed mice. Lab Invest.

[B46] Ku NO, Liao J, Omary MB (1998). Phosphorylation of human keratin 18 serine 33 regulates binding to 14-3-3 proteins. Embo J.

[B47] Ku NO, Michie S, Resurreccion EZ, Broome RL, Omary MB (2002). Keratin binding to 14-3-3 proteins modulates keratin filaments and hepatocyte mitotic progression. Proc Natl Acad Sci U S A.

[B48] Ku NO, Omary MB (1997). Phosphorylation of human keratin 8 in vivo at conserved head domain serine 23 and at epidermal growth factor-stimulated tail domain serine 431. J Biol Chem.

[B49] Stumptner C, Omary MB, Fickert P, Denk H, Zatloukal K (2000). Hepatocyte cytokeratins are hyperphosphorylated at multiple sites in human alcoholic hepatitis and in a mallory body mouse model. Am J Pathol.

[B50] Liao J, Lowthert LA, Ghori N, Omary MB (1995). The 70-kDa heat shock proteins associate with glandular intermediate filaments in an ATP-dependent manner. J Biol Chem.

[B51] Ku NO, Omary MB (2000). Keratins turn over by ubiquitination in a phosphorylation-modulated fashion. J Cell Biol.

[B52] Gabai VL, Meriin AB, Mosser DD, Caron AW, Rits S, Shifrin VI, Sherman MY (1997). Hsp70 prevents activation of stress kinases. A novel pathway of cellular thermotolerance. J Biol Chem.

[B53] Bardag-Gorce F, van Leeuwen FW, Nguyen V, French BA, Li J, Riley N, McPhaul LW, Lue YH, French SW (2002). The role of the ubiquitin-proteasome pathway in the formation of mallory bodies. Exp Mol Pathol.

[B54] Stumptner C, Fuchsbichler A, Lehner M, Zatloukal K, Denk H (2001). Sequence of events in the assembly of Mallory body components in mouse liver: clues to the pathogenesis and significance of Mallory body formation. J Hepatol.

[B55] He T, Stepulak A, Holmstrom TH, Omary MB, Eriksson JE (2002). The intermediate filament protein keratin 8 is a novel cytoplasmic substrate for c-Jun N-terminal kinase. J Biol Chem.

[B56] Boller K, Kemler R, Baribault H, Doetschman T (1987). Differential distribution of cytokeratins after microinjection of anti-cytokeratin monoclonal antibodies. Eur J Cell Biol.

[B57] Laemmli UK (1970). Cleavage of structural proteins during the assembly of the head of bacteriophage T4. Nature.

[B58] Lowry OH, Rosebrough NJ, Farr AL, Randall RJ (1951). Protein measurement with the Phenol folin reagent. J Biol Chem.

